# In vitro anti-allergic activity of *Moringa oleifera* Lam. extracts and their isolated compounds

**DOI:** 10.1186/s12906-019-2776-1

**Published:** 2019-12-11

**Authors:** Nur Zahirah Abd Rani, Endang Kumolosasi, Malina Jasamai, Jamia Azdina Jamal, Kok Wai Lam, Khairana Husain

**Affiliations:** Drug and Herbal Research Centre, Faculty of Pharmacy, Universiti Kebangsaan Malaysia, Jalan Raja Muda Abdul Aziz, 50300 Kuala Lumpur, Malaysia

**Keywords:** *Moringa oleifera*, Anti-allergic, Isolation, RBL-2H3, Histamine, Beta-hexosaminidase, IL-4, TNF- α, New compound

## Abstract

**Background:**

*Moringa oleifera* Lam. is a commonly used plant in herbal medicine and has various reported bioactivities such as antioxidant, antimicrobial, anticancer and antidiabetes. It is rich in nutrients and polyphenols. The plant also has been traditionally used for alleviating allergic conditions. This study was aimed to examine the anti-allergic activity of *M. oleifera* extracts and its isolated compounds.

**Method:**

*M. oleifera* leaves, seeds and pods were extracted with 80% of ethanol. Individual compounds were isolated using a column chromatographic technique and elucidated based on the nuclear magnetic resonance (NMR) and electrospray ionisation mass spectrometry (ESIMS) spectral data. The anti-allergic activity of the extracts, isolated compounds and ketotifen fumarate as a positive control was evaluated using rat basophilic leukaemia (RBL-2H3) cells for early and late phases of allergic reactions. The early phase was determined based on the inhibition of beta-hexosaminidase and histamine release; while the late phase was based on the inhibition of interleukin (IL-4) and tumour necrosis factor (TNF-α) release.

**Results:**

Two new compounds; ethyl-(*E*)–undec-6-enoate (**1**) and 3,5,6-trihydroxy-2-(2,3,4,5,6-pentahydroxyphenyl)-4*H*-chromen-4-one (**2**) together with six known compounds; quercetin (**3**), kaempferol (**4**), β-sitosterol-3-*O*-glucoside (**5**), oleic acid (**6**), glucomoringin (**7**), 2,3,4-trihydroxybenzaldehyde (**8**) and stigmasterol (**9**) were isolated from *M. oleifera* extracts. All extracts and the isolated compounds inhibited mast cell degranulation by inhibiting beta-hexosaminidase and histamine release, as well as the release of IL-4 and TNF-α at varying levels compared with ketotifen fumarate.

**Conclusion:**

The study suggested that *M. oleifera* and its isolated compounds potentially have an anti-allergic activity by inhibiting both early and late phases of allergic reactions.

## Background

*Moringa oleifera* Lam. (*M. oleifera*) is well known for its nutritional and medicinal values [1]. It is mainly cultivated in the tropical and subtropical continents due to its high tolerance to extreme weather and as a nutritional source [2–4]. The plant is also commonly known as kelor, drumstick tree, horseradish tree, marunggay and Sajna [1, 5].

*M. oleifera* has been reported to contain seven times more vitamin C than oranges, four times more vitamin A than carrots, four times more calcium than milk, two times more protein than yogurt and three times more potassium than bananas [6]. Its immature pods, flowers and leaves contain high percentage of essential amino acids [7]. The whole plant has high content of unsaturated and essential fatty acid such as linolenic acid, linoleic acid and oleic acid; as well as micronutrients such as iron, zinc, vitamin A, calcium and β-carotene [8]. The ability of the nutritious plant to rapidly grow in harsh climates with high yield helps in combatting severe malnutrition of the populations in the subtropical and tropical regions [9].

*M. oleifera* also contains high content of polyphenols that contributes to its high total antioxidant capacity and could be medicinally useful for diseases such as cancer, hypertension and hyperglycaemia [8, 10]. The plant has been reported to have antimicrobial activity against waterborne [11], oral [12] and coliform [13] bacteria, as well as dermatophytes [14]. It has anti-diabetic and anti-inflammatory activities that could be associated with high isothiocyanates content [15–17]. Its polyphenols such as chlorogenic acid, vicenin-2 and quercetin were also found to contribute to the wound healing activity [18].

Allergic reaction cases are increasing by years. The reactions can be as mild as a rash and as severe as an anaphylactic shock that can lead to death. Subsequent exposure of allergens will trigger mast cell degranulation, releasing mediators that exhibit allergic symptoms. Early phase of an allergic reaction usually occurs minutes after sensitisation, triggering the release of histamine, that will induce bronchoconstriction, vasodilation and vascular permeability [19]. Meanwhile, the late phase of an allergic reaction occurring hours after sensitisation will trigger the release of cytokines such as IL-4, IL-5, IL-9, IL-13, TNF-α and IFN-γ that will recruit inflammatory cells and further bring about mast cell degranulation.

Ayurvedic practitioners have been using *M. oleifera* to alleviate allergic conditions but scientific evidences are still lacking [20]. Due to the high content of flavonoids, the plant could be associated with the anti-allergic activity via inhibition of histamine and IL-4 release [10, 21]. In this study, the anti-allergic activity of *M. oleifera* extracts from various plant parts and its isolated compounds was evaluated using basophil cells to determine the inhibition of mast cell degranulation and cytokines production.

## Methods

### Materials and equipment

Pre-coated aluminium plate of thin layer chromatography (TLC) with silica gel 60 GF254 of 20 × 20 cm dimension (Merck, Darmstadt, Germany) was used to visualize separated spots of compounds upon chromatographic development under ultraviolet light (λ_254_, λ_366_, model UVGL-58). The compounds isolation process was conducted using a column chromatographic technique with silica gel 60 (230–400 mesh ASTM) (Merck, Darmstadt, Germany) and Sephadex LH20 (GE Healthcare, Uppsala, Sweden) as the stationary phases. Chemical structures of the compounds were elucidated using a nuclear magnetic resonance (NMR) spectrometer (^1^H NMR 600 MHz and ^13^C NMR 151 MHz, Cryoprobe, Bruker, Basel, Switzerland). Mass of the compounds was analysed using a high-resolution electrospray ionization mass spectrometer (HRESIMS) (Micro TOF-Q, Bruker, Basel, Switzerland). A 24 well-plate, a 96 well-plate and a 75cm^2^ cell culture flask were obtained from NEST, China. An incubator (IR 230 Forma, Thermo Scientific, Massachusetts, USA) and an ELISA microplate reader (Tecan Infinite M200 Pro, Mannedorf, Switzerland) were used throughout the bioassays studies.

### Chemicals and reagents

The TLC plate was sprayed with 10% sulfuric acid and Dragendorff reagents to visualise the separated spots of compounds obtained. Organic solvents used for the isolation of compounds were of analytical grade (Merck, Darmstadt, Germany). Deuterated chloroform (CDCl_3_) and methanol (MeOD) were used in the NMR spectroscopy. Ketotifen fumarate (Abcam, Cambridge, United Kingdom) was used as a positive control for the bioassay experiments. Minimum essential media (MEM) with Earle’s salts and *L*-glutamine, foetal bovine serum (FBS) from Biowest (Missouri, USA) and penicillin-streptomycin (10,000 U/mL) (Gibco, New Hampshire, USA) were used as cell media.

Rat basophilic leukemic (RBL)-2H3 cells were obtained from the Japanese Collection of Research Bioresources Cell Bank (JCRB, Japan). Dimethyl sulfoxide (DMSO) (Merck, New Jersey, USA) was used for dissolving test samples. The concentration of DMSO was kept below 0.25% as it was reported safe for RBL-2H3 cell [22]. 3-(4,5-Dimethylthiazol-2-yl)-2,5-diphenyltetrazolium bromide (MTT) (Goldbio Technology, Missouri, USA) was used to determine cell viability. Monoclonal Anti-Dinitrophenyl (DNP) IgE antibody produced in mouse (Sigma-Aldrich, New Hampshire, USA) were used for cell sensitisation. Bovine serum albumin (BSA) solution (Sigma-Aldrich, New Hampshire, USA), dinitrophenyl-bovine serum albumin (DNP-BSA) (Santa Cruz, USA) and *p*-nitrophenyl-*N*-acethyl-β-D-glucosaminide (Merck, New Jersey, USA) were used for the bioassays. Histamine, TNF-α and IL-4 Elisa kits were obtained from Elabscience, China.

### Plant materials

Leaves, seeds and pods of *M. oleifera* were obtained from Terengganu, Malaysia. The taxonomy of the specimens was confirmed by Dr. Shamsul Khamis, a botanist from Universiti Kebangsaan Malaysia (UKM). The specimens were deposited at the Universiti Kebangsaan Malaysia Herbarium in Bangi with a voucher specimen UKMB40408.

### Preparation of extracts and isolation of compounds

The plant materials were air-dried and ground before the extraction process. The leaves (848.9 g), seeds (1999.2 g) and pods (2000.0 g) were individually macerated with 80% of ethanol for three days and filtered. The residue was extracted twice. Filtrates of each plant part were collected and evaporated using a rotary evaporator. The remaining water content was removed using a freeze-drying technique. The yield of leaf, seed and pod extracts were 42.43 g, 65.05 g and 59.56 g respectively. The extracts were sequentially fractionated using hexane, ethyl acetate and acetone as eluting solvents with various compositions.

### Structural elucidation of isolated compounds

#### Ethyl-(*E*)-undec-6-enoate (1)

Elution of the hexane leaf fraction (18.91 g) with hexane-ethyl acetate (8:2, v/v) yielded compound **1**. White amorphous liquid (34.2 mg), TLC: R_f_ 0.83 (hexane: EtOAc, 8:2), HRESIMS (+ve mode) *m/z*: 235.1295 [M + Na]^+^(calculated for C_13_H_24_O_2,_ 212.33 g/mol). ^1^H NMR (CDCl_3_, 600 MHz): δ_H_ 0.85 (t, 3H, *J* = 7.1 Hz, H-13), 1.25 (m, 21H, H-1, H-12), 1.32 (m, 21H, H-6), 1.35 (m, 21H, H-11), 1.62 (m, 2H, H-5), 2.02 (m, 2H, H-10), 2.31 (t, 2H, *J* = 7.7 Hz, H-4), 2.79 (m, 1H, H-7), 4.15 (q, 2H, *J* = 7.1 Hz, H-2), 5.4 (m, 1H, H-8, H-9). ^13^C NMR (CDCl_3_, 150 MHz): δc 14.3 (C-1, C-13), 22.8 (C-12), 24.9 (C-6), 29.1 (C-5), 29.7 (C-11), 31.9 (C-10), 35 (C-4, C7), 60.1 (C-2), 130 (C-8, C-9), 174.3 (C-3).

#### 3,5,6-trihydroxy-2-(2,3,4,5,6-pentahydroxyphenyl)-4*H*-chromen-4-one (2)

The ethyl acetate leaf fraction (3.38 g) was loaded onto Sephadex LH-20 using methanol-water (1:1, v/v) yielding four fractions. The third fraction was further eluted using Sephadex LH-20 with chloroform-dichloromethane-methanol (4:1:1, v/v) as a mobile phase giving four fractions. The last fraction was further eluted in silica gel column chromatography with ethyl acetate-chloroform (7:3, v/v) as a mobile phase yielding compound **2**. Reddish-brown liquid (3.9 mg), TLC: R_f_ 0.50 (CHCl_3_: MeOH, 9:1), HRESIMS (+ve mode) *m/z*: 176.9835 [M + 2H]^2+^(calculated for C_15_H_10_O_10,_ 350.24 g/mol) .^1^H NMR (CDCl_3_, 600 MHz): δ_H_ 6.83 (d, 1H, *J* = 8.6 Hz, H-7), 7.89 (d, 1H, *J* = 8.7 Hz, H-8). ^13^C NMR (CDCl_3_, 150 MHz): δ_c_ 113.3 (C-10), 114.3 (C-7), 122.1 (C-1′), 123.0 (C-6), 128.5 (C-3′), 128.5 (C-5′), 131.4 (C-8), 138.5 (C-3), 138.5 (C-4′), 147.5 (C-2′), 147.5 (C-6′), 161.8 (C-9), 162.9 (C-5), 168.9 (C-2), 173.8 (C-4).

#### Quercetin (3)

The ethyl acetate leaf fraction (3.38 g) was loaded onto Sephadex LH-20 using methanol-water (1:1, v/v) yielding four fractions. The fourth fraction was further eluted using Sephadex LH-20 with chloroform-dichloromethane-methanol (4:1:1, v/v) as a mobile phase yielding compound **3**. Yellow powder (5.9 mg), m.p 312–314 °C. TLC: R_f_ 0.49 (CHCl_3_: MeOH, 9:1), HRESIMS (+ve mode) *m/z*: 325.0294 [M + Na]^+^(calculated for C_15_H_10_O_7,_ 302.24 g/mol). ^1^H NMR (CDCl_3_, 600 MHz): δ_H_ 6.20 (d, 1H, *J* = 1.8 Hz, H-6), 6.41 (d, 1H, *J* = 1.8 Hz, H-8), 6.91 (d, 1H, *J* = 8.4 Hz, H-5′), 7.66 (dd, 1H, *J* = 8.4 Hz, H-6′), 7.76 (d, 1H, *J* = 2.4 Hz, H-2′). ^13^C NMR (CDCl_3_, 150 MHz): δ_c_ 93.3 (C-8), 98.2 (C-6), 103.3 (C-10), 114.9 (C-2′), 115.1 (C-5′), 120.6 (C-6′), 123.0 (C-1′), 136.1 (C-3), 145.1 (C-3′), 146.9 (C-2), 147.6 (C-4′), 157.1 (C-9), 161.3 (C-5), 164.5 (C-7), 176.2 (C-4).

#### Kaempferol (4)

The ethyl acetate leaf fraction (3.38 g) was loaded onto Sephadex LH-20 using methanol-water (1:1, v/v) yielding four fractions. The fourth fraction was further eluted using Sephadex LH-20 with chloroform-dichloromethane-methanol (4:1:1, v/v) as a mobile phase giving another six fractions. The fifth fraction was subjected to silica gel column chromatography using chloroform-methanol (9:1, v/v) as a mobile phase to obtain compound **4**. Yellow powder (1.3 mg), m.p 276–279 °C. TLC: R_f_ 0.68 (CHCl_3_: MeOH, 9:1), HRESIMS (+ve mode) *m/z*: 309.0334 [M + Na]^+^(calculated for C_15_H_10_O_6_, 286.24 g/mol). ^1^H NMR (CDCl_3_, 600 MHz): δ_H_ 6.19 (s, 1H, H-6), 6.41 (s, 1H, H-8), 6.91 (d, 1H, *J* = 9.0 Hz, H-3′), 6.91 (d, 1H, *J* = 9.0 Hz, H-5′), 8.10 (d, 1H, *J* = 8.4 Hz, H-6′), 8.74 (s, 1H, H-2′).^13^C NMR (CDCl_3_, 150 MHz): δ_c_ 93.1 (C-8), 97.9 (C-6), 103.2 (C-10), 114.9 (C-3′), 114.9 (C-5′), 122.3 (C-1′), 129.3 (C-2′), 129.3 (C-6′), 135.7 (C-3), 146.7 (C-2), 156.9 (C-9), 159.2 (C-4′), 161.1 (C-5), 164.2 (C-7), 176.0 (C-4).

#### β-Sitosterol-3-*O*-glucoside (5)

The acetone leaf fraction (0.95 g) was eluted in the silica gel column chromatography using ethyl acetate-chloroform (7:3, v/v) as a mobile phase to obtain seven fractions. The sixth fraction was separated using ethyl acetate (8:2, v/v) yielding four fractions. The fourth fraction was then purified using chloroform-methanol (9:1, v/v) to give compound **5**. White powder (8.5 mg), m.p 209–211 °C. TLC: R_f_ 0.67 (CHCl_3_: MeOH, 9:1), HRESIMS (+ve mode) *m/z*: 599.4190 [M + Na]^+^ (calculated for C_35_H_60_O_6,_ 576.86 g/mol). ^1^H NMR (CDCl_3_, 600 MHz): δ_H_ 0.68 (s, 3H, H-18), 0.83 (m, 7H, H-9), 0.83 (m, 7H, H-14), 0.83 (m, 7H, H-24), 0.83 (m, 7H, H-29), 0.92 (d, 4H, *J* = 6.6 Hz, H-21), 0.92 (d, 4H, *J* = 6.6 Hz, H-26), 0.92 (d, 4H, *J* = 6.6 Hz, H-27), 0.96 (s, 6H, H-19), 1.00 (m, 6H, H-1), 1.00 (m, 6H, H-15), 1.17 (m, 5H, H-12), 1.17 (m, 5H, H-17), 1.25 (m, 4H, H-22), 1.25 (m, 4H, H-23), 1.25 (m, 4H, H-28), 1.37 (m, 6H, H-8), 1.38 (m, 6H, H-20), 1.42 (m, 6H, H-11), 1.52 (m, 5H, H-2), 1.52 (m, 5H, H-15), 1.53 (m, 5H, H-12), 1.65 (m, 3H, H-7), 1.65 (m, 3H, H-25), 1.82 (m, 4H, H-16), 1.95 (m, 4H, H-7), 1.98 (m, 4H, H-4), 2.63 (m, 1H, H-3), 2.90 (m, 2H, H-2′), 3.00 (td, 2H, *J* = 5.2 Hz, H-4′), 3.08 (dd, 2H, *J* = 2 Hz, H-5′), 3.12 (td, 2H, *J* = 4.8 Hz, H-3′), 3.48 (m, 2H, H-2′(OH)), 3.48 (m, 2H, H-3′(OH)), 3.48 (m, 2H, H-4′(OH)), 3.68 (m, 2H, H-6′(OH)), 4.25 (d, 1H, *J* = 7.8 Hz, H-6′), 4.45 (d, 1H, *J* = 5.8 Hz, H-6′), 4.91 (d, 3H, *J* = 6.1 Hz, H-1′), 5.35 (m, 1H, H-6). ^13^C NMR (CDCl_3_, 150 MHz): δ_c_ 12.2 (C-18), 12.3 (C-29), 19.1 (C-21), 19.4 (C-27), 19.6 (C-19), 20.2 (C-26), 23.1 (C-28), 24.3 (C-15), 25.9 (C-23), 28.3 (C-16), 29.1 (C-25), 29.7 (C-2), 31.8 (C-7), 31.8 (C-8), 31.8 (C-22), 36.0 (C-10), 36.0 (C-20), 36.7 (C-1), 37.3 (C-12), 38.8 (C-4), 42.3 (C-13), 45.6 (C-24), 50.0 (C-9), 55.9 (C-17), 56.6 (C-14), 61.6 (C-6′), 70.6 (C-4′), 73.9 (C-2′), 77.2 (C-5′), 77.3 (C-3), 77.3 (C-3′), 101.2 (C-1′), 121.7 (C-6), 140.9 (C-5).

#### Oleic acid (6)

The ethyl acetate seed fraction (2.10 g) was eluted in the silica gel column chromatography using hexane-ethyl acetate (7:3, v/v) as a mobile phase yielding three fractions. The second fraction was further eluted using hexane-ethyl acetate (8:2, v/v) yielding compound **6**. White amorphous liquid (24.5 mg), TLC: R_f_ 0.74 (hexane: EtOAc, 7:3), HRESIMS (+ve mode) *m/z*: 603.4867 [2 M + K]^+^ (calculated for C_18_H_34_O_2,_ 282.47 g/mol). ^1^H NMR (CDCl_3_, 600 MHz): δ_H_ 0.90 (t, 3H, *J* = 7.2 Hz, H-18), 1.30 (m, 20H, H-4, H-5, H-6, H-7, H-12, H-13, H-14, H-15, H-16, H-17), 1.65 (m, 2H, H-3), 2.03 (m, 1H, H-8), 2.03 (m, 1H, H-11), 2.37 (t, 2H, *J* = 7.2 Hz, H-2), 5.37 (m, 1H, H-9), 5.37 (m, 1H, H-9). ^13^C NMR (CDCl_3_, 150 MHz): δ_c_ 14.1 (C-18), 22.7 (C-17), 24.7 (C-3), 27.2 (C-8), 27.2 (C-11), 29.0 (C-4), 29.0 (C-5), 29.1 (C-14), 29.1 (C-15), 29.4 (C-13), 29.5 (C-6), 29.7 (C-12), 29.8 (C-7), 31.9 (C-16), 34.3 (C-2), 129.8 (C-10), 130.0 (C-9), 179.8 (C-1).

#### Glucomoringin (7)

The acetone seed fraction (2.21 g) was eluted in the silica gel column chromatography using chloroform-methanol (9:1, v/v) as a mobile phase yielding five fractions. The fourth fraction was eluted using the same mobile phase to obtain compound **7**. Yellow liquid (28.1 mg), TLC: R_f_ 0.36 (CHCl_3_: MeOH, 9:1), HRESIMS (+ve mode) *m/z*: 572.3460 [M + H]^+^ (calculated for C_20_H_29_NO_14_S_2,_ 571.57 g/mol). ^1^H NMR (CDCl_3_, 600 MHz): δ_H_ 1.23 (q, 4H, *J* = 4.8 Hz, H-6″), 3.20 (m, H-6′), 3.49 (m, H-4″), 3.64 (m, 1H, H-5″), 3.83 (s, 1H, H-7), 3.86 (dt, 1H, *J* = 1.8, 8.7 Hz, H-3″), 4.03 (m, 1H, H-2″), 4.10 (m, H-5′), 4.23 (m, H-4′), 4.34 (m, H-3′), 4.47 (d, 1H, *J* = 7.2 Hz, H-1′), 4.64 (m, H-2′), 5.44 (dd, 1H, *J* = 1.8, 17.4 Hz, H-1′), 7.02 (d, 1H, *J* = 4.8 Hz, H-1), 7.09 (d, 1H, *J* = 4.8 Hz, H-6), 7.27 (d, 1H, *J* = 3 Hz, H-5), 7.30 (d, 1H, *J* = 8.4 Hz, H-3). ^13^C NMR (CDCl_3_, 150 MHz): δ_c_ 16.7 (C-6″), 21.2 (C-7), 43.0 (C-4′), 45.5 (C-3′), 47.1 (C-2′), 47.9 (C-6′), 59.8 (C-5′), 65.9 (C-1′), 69.2 (C-5″), 69.3 (C-2″), 70.6 (C-3″), 72.3 (C-4″), 98.4 (C-1″), 116.1 (C-6), 116.7 (C-2), 128.7 (C-3), 129.0 (C-5), 131.7 (C-4), 155.7 (C-1), 156.0 (N).

#### 2,3,4-trihydroxybenzaldehyde (8)

The hexane pod fraction (19.2 g) was eluted on the silica gel column chromatography using hexane-ethyl acetate (8:2, v/v) as a mobile phase yielding four fractions. The second fraction was further eluted using hexane-ethyl acetate (9:1, v/v) giving five fractions. The third fraction was purified using hexane-ethyl acetate (7:3, v/v) yielding compound **8**. Reddish brown liquid (1.8 mg), TLC: R_f_ 0.73 (CHCl_3_: MeOH, 9:1), HRESIMS (+ve mode) *m/z*193.0489[M + K] ^+^(calculated for C_7_H_6_O_4,_ 154.12 g/mol). ^1^H NMR (CDCl_3_, 600 MHz): δ_H_ 6.98 (d, 1H, *J* = 12 Hz, H-5), 7.83 (d, 1H, *J* = 12 Hz, H-6), 9.89 (s, 1H, H-7). ^13^C NMR (CDCl_3_, 150 MHz): δ_c_ 116.0 (C-1), 116.0 (C-5), 130.2 (C-3), 132.4 (C-2), 132.4 (C-4), 161.2 (C-6), 190.8 (C-7).

#### Stigmasterol (9)

The ethyl acetate pod fraction (3.0 g) was eluted in the silica gel column chromatography using chloroform-ethyl acetate (6:4, v/v) as a mobile phase eluting fourteen fractions. The second fraction was further subjected to silica gel column chromatography using hexane-ethyl acetate (8:2, v/v) to obtain compound **9**. White crystal (28.1 mg), m.p 166–168 °C. TLC: R_f_ 0.39 (Hexane: EtOAc, 9:1), HR ESIMS (+ve mode) *m/z*: 451.3425 [M + K] ^+^ (calculated for C_29_H_48_O, 412.70 g/mol). ^1^H NMR (CDCl_3_, 600 MHz): δ_H_ 0.70 (s, 1H, H-18), 0.82 (m, 3H, H-27), 0.83 (m, 3H, H-29), 0.87 (m, 3H, H-26), 0.95 (m, 3H, H-21), 0.95 (m, 3H, H-9), 1.02 (s, 3H, H-19), 1.05 (m, 2H, H-14), 1.08 (m, 1H, H-15), 1.12 (m, 2H, H-1), 1.18 (m, 2H, H-17), 1.28 (s, 1H, H-12), 1.37 (m, 2H, H-28), 1.46 (m, 1H, H-8), 1.48 (m, 2H, H-11), 1.49 (m, 1H, H-2), 1.52 (m, 1H, H-24), 1.53 (m, 1H, H-25), 1.55 (m, 1H, H-15), 1.58 (m, 2H, H-16), 1.71 (m, 1H, H-2), 1.85 (m, 1H, H-1), 1.88 (m, 2H, H-7), 1.98 (m, 1H, H-12), 2.25 (m, 1H, H-20), 2.32 (m, 1H, H-4), 3.55 (m, 1H, H-3), 5.04 (dd, 1H, *J* = 15 Hz, H-23), 5.16 (dd, 1H, *J* = 18 Hz, H-22), 5.38 (m, 1H, H-6). ^13^C NMR (CDCl_3_, 150 MHz): δ_c_ 12.0 (C-18), 12.3 (C-29), 19.4 (C-19), 19.9 (C-27), 21.1 (C-11), 21.1 (C-26), 21.3 (C-22), 24.3 (C-15), 25.6 (C-28), 29.0 (C-16), 29.8 (C-2), 31.9 (C-7), 31.9 (C-8), 31.9 (C-25), 36.5 (C-10), 37.8 (C-1), 39.8 (C-12), 40.0 (C-20), 42.3 (C-13), 44.0 (C-4), 50.1 (C-9), 51.2 (C-24), 55.9 (C-17), 56.8 (C-14), 71.8 (C-3), 121.8 (C-6), 129.3 (C-23), 138.3 (C-22), 140.8 (C-5).

### Cell viability assay

RBL-2H3 cells from the Japanese Collection of Research Bioresources Cell Bank (JCRB, Japan) in enriched media (100 μL) were seeded in 96 well plates with cell concentrations of 2.5 × 10^5^ cells/mL. The cells were incubated for 24 h at 37 °C in a humidified incubator (5% CO_2_, 95% air) prior to reconstitution with 100 μL of test sample. Then the cells were incubated for another 24 h. 10 μL of 3-(4,5-dimethylthiazol-2-yl)-2,5-diphenyltetrazolium bromide (MTT) (5 mg/mL in PBS buffer) was added into the mixture and was incubated further for 4 h. Upon the appearance of purple formazan crystal, the medium was removed and 100 μL of DMSO was added. After shaking the plates for 10 min, the absorbance was immediately measured using a microplate reader at 570 nm. Percentage of cell viability was determined as using the formula below, whereby OD means optical density:

$$ \mathrm{Percentage}\ \mathrm{of}\ \mathrm{cell}\ \mathrm{viability}\ \left(\%\right)=\frac{\ \mathrm{OD}\ \mathrm{test}\ \mathrm{sample}-\mathrm{OD}\ \mathrm{blank}\kern0.5em }{\mathrm{OD}\ \mathrm{control}-\mathrm{OD}\ \mathrm{blank}}\ \mathrm{x}\ 100 $$

### Preparation of RBL-2H3 cells for degranulation assessment

RBL-2H3 cells were cultured in MEM containing 10% FBS and 1% penicillin-streptomycin at 37 °C with 5% CO_2_ and 95% air. The medium was changed every two days and the cells were sub-cultured upon 80% confluency. Once the cells reached the third passage with stable morphology and growth, the cells were used for the bioassay experiment. Only cells from third to fifth passages were used in this study.

RBL-2H3 cells (2 × 10^5^ / 400 μL) in enriched medium were seeded and allowed to adhere onto 24 well plates in the humidified incubator (37 °C, 5% CO_2_, 95% air) for two hours. The cells were then sensitized with anti-DNP IgE (0.45 μg/mL, 100 μL) overnight at 37 °C in 5% CO_2_.

### Inhibitory effect of beta-hexosaminidase release

The inhibitory effect of beta-hexosaminidase release from RBL-2H3 cells were measured using the method described by Shahari et al. [23]. The sensitised cells were washed twice with 500 μL Siraganian buffer (119 mM NaCl, 5 mM KCl, 5.6 mM glucose, 0.4 mM MgCl_2_, 1 mM CaCl_2_, 25 mM piperazine-*N*, *N*′-bis (2-ethanesulfonic acid) (PIPES), 40 mM NaOH, 0.1% BSA, pH 7.2) and were reconstituted with 160 μL Siraganian buffer. After 10 min of incubation at 37 °C in 5% CO_2_ and 95% air, the cells were treated with 20 μL of test samples (7.81, 15.62 and 31.25 μg/mL) or 20 μL of ketotifen fumarate (7.81, 15.62 and 31.25 μg/mL) and incubated for another 10 min. Cells degranulation was then stimulated by the addition of 20 μL DNP-BSA (10 mg/mL) allergen for 30 min at 37 °C in 5% CO_2_ and 95% air. Aliquot of the supernatant (50 μL) was then transferred into a 96 well plate and incubated with a substrate (1 μM *p*-nitrophenyl-N-acetyl-β-D-glucosaminide in 0.1 M citrate buffer, pH 4.5) in 1:1 ratio at 37 °C for 1 h. The reaction was quenched with the addition of 200 μL of stop solution (0.1 M Na_2_CO_3_/ NaHCO_3_, pH 10.0). The absorbance was measured at 405 nm using a microplate reader. Cells that were exposed to allergen were used as negative control. While cells that did not exposed to allergen were used as normal. The positive control used was ketotifen fumarate. The percentage inhibition was determined using the equation below.

$$ \mathrm{Percentage}\ \mathrm{inhibition}\ \left(\%\right)=\left(1-\kern0.5em \frac{\ \mathrm{OD}\ \mathrm{test}\ \mathrm{sample}-\mathrm{OD}\ \mathrm{blank}-\mathrm{OD}\ \mathrm{normal}\ }{\mathrm{OD}\ \mathrm{control}-\mathrm{OD}\ \mathrm{normal}}\ \right)\ \mathrm{x}\ 100 $$

### Inhibitory effects on histamine and cytokines release

The inhibitory effects on histamine and cytokines release from RBL-2H3 cells were measured using the previously described methods [24, 25]. The sensitised cells were washed twice with 500 μL enriched medium and were reconstituted with 320 μL of enriched medium. After 10 min of incubation, the cells were treated with 40 μL of test samples (7.81, 15.62 and 31.25 μg/mL) or 40 μL of ketotifen fumarate (7.81, 15.62 and 31.25 μg/mL). For early-phase allergic reaction assay, the cells were stimulated using 40 μL of DNP-BSA (10 mg/mL) allergen for 30 min at 37 °C in 5% CO_2_ and 95% air. Aliquot of the supernatant (50 μL) was then centrifuged at 1000 x g at 8 °C for 20 min and the concentration of histamine released was measured using a Histamine ELISA Kit (Elabscience, China). For late-phase allergic reaction assay, the cells were incubated with 40 μL of DNP-BSA (10 mg/mL) allergen for 4 h at 37 °C in 5% CO_2_ and 95% air. The supernatant (100 μL) was centrifuged at 1000 x g at 8 °C for 20 min and the concentrations of IL-4 and TNF-α were measured using the respective ELISA kits (Elabscience, China).

### Statistical analysis

The data obtained were reported as mean value ± standard error of mean (SEM) representing triplicate measurements (*n* = 3). The IC_50_ values with 95% confidence intervals were determined using a GraphPad Prism 5 software. Statistical significance of the data (*p*-value < 0.05) was analysed using one-way ANOVA with GraphPad Prism 5.

## Results

### Structure elucidation

**Fig. 1:**
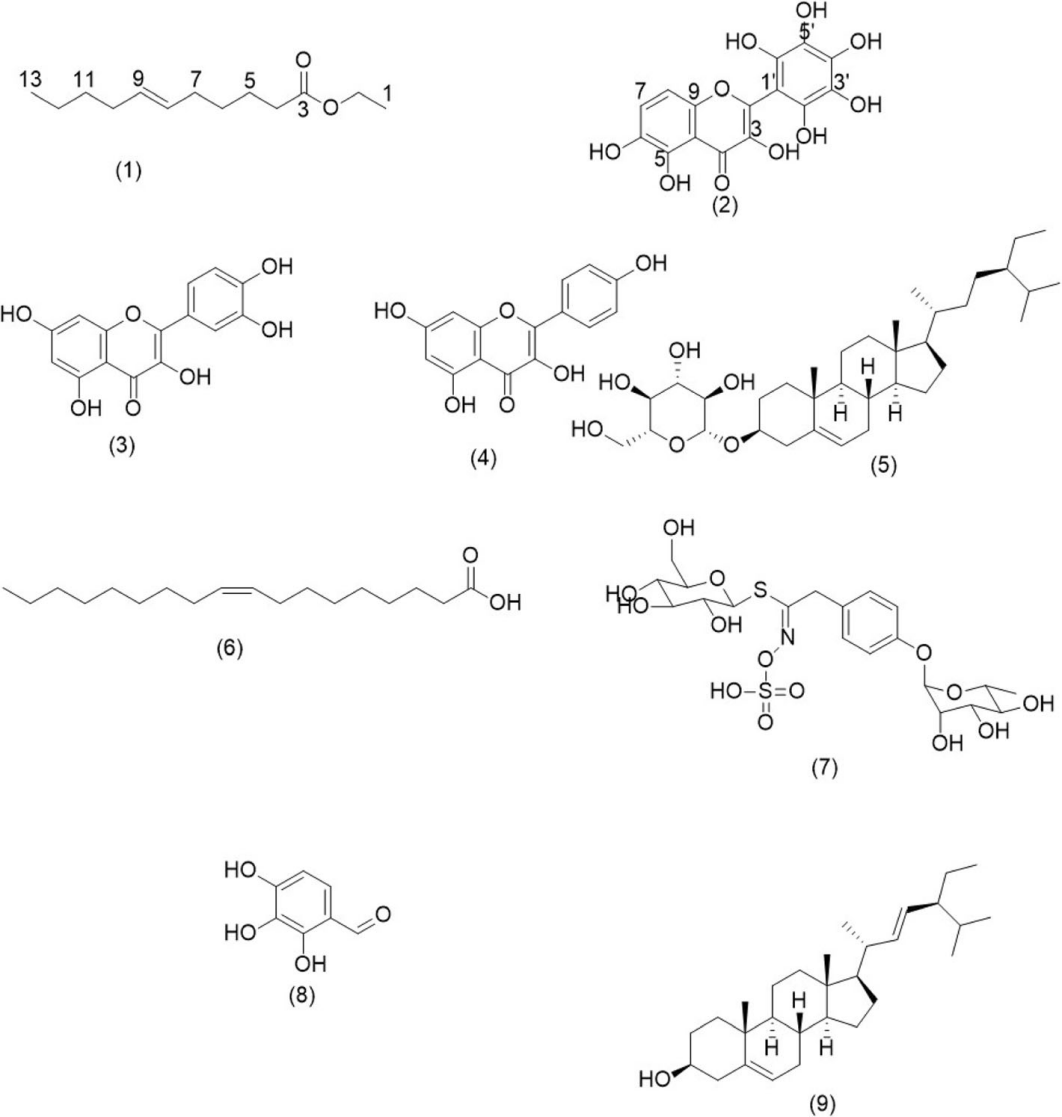
Chemical structure of isolated compounds from *M. oleifera*. Ethyl-(*E*)-undec-6-enoate (1), 3,5,6-trihydroxy-2-(2,3,4,5,6-pentahydroxyphenyl)-4*H*-chromen-4-one (2), Quercetin (3), Kaempferol (4), β-sitosterol-3-*O*-glucoside (5), Oleic acid (6), Glucomoringin (7), Stigmasterol (9)

**Fig. 2:**
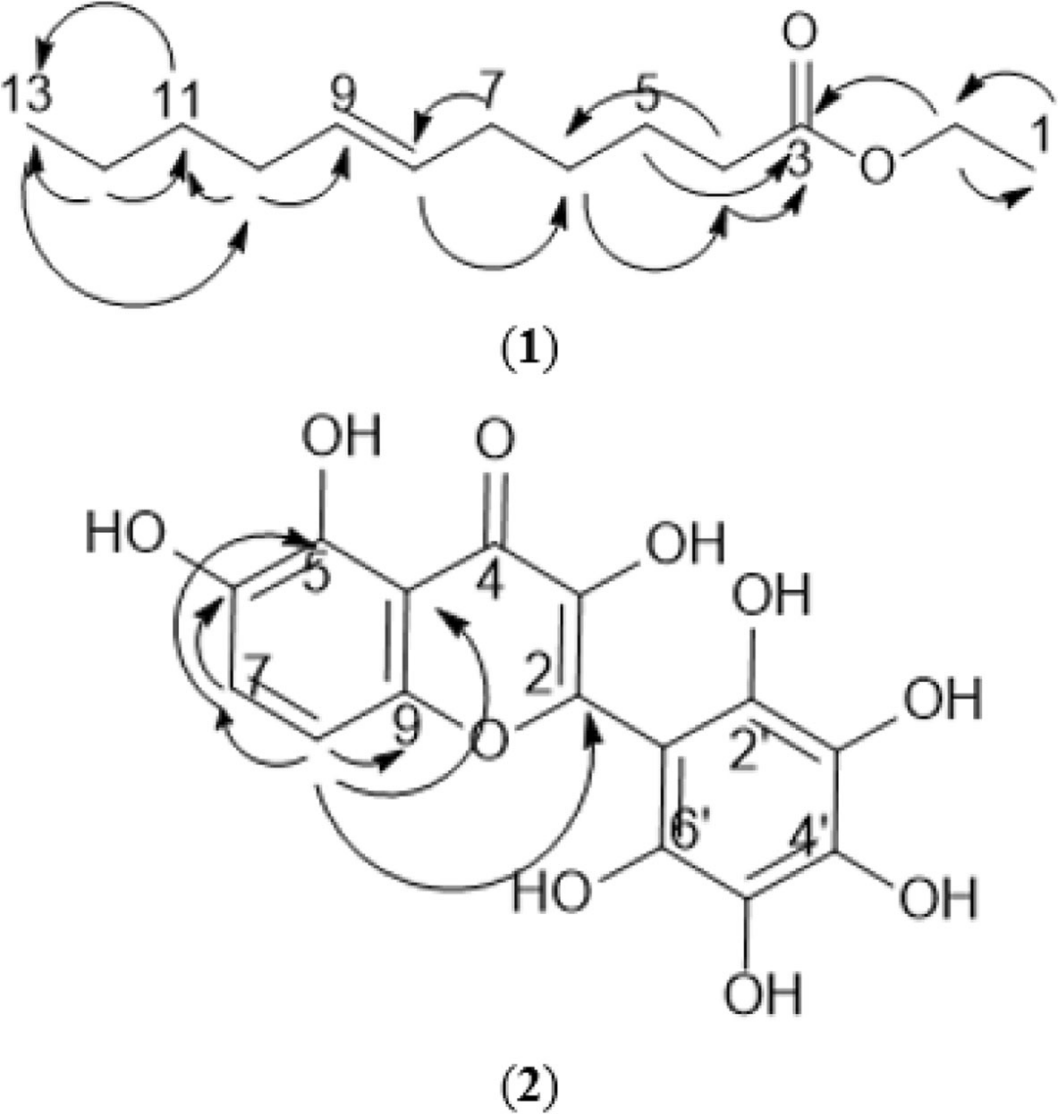
HMBC of new isolated compounds (1 and 2)

The elution of hexane fraction of *M. oleifera* leaf extract yielded a new compound (compound **1)**. Compound **1** was obtained as white amorphous powder (34.2 mg) with HRESIMS molecular ionisation at *m/z* 235.1295 corresponding to [M + Na] ^+^. Based on the value, chemical formula of the compound was deduced as C_13_H_24_O_2_ with a molecular weight of 212.33 g/mol. ^1^H and ^13^C NMR spectral data of the compound (Table 1) suggest the presence of a long chain skeleton with an olefinic signal at δ 5.41 (H-8, H-9) and one carbonyl signal at δ 174.13 (C-3). The carbonyl group was positioned at C-3 and the olefin at H-8 as evidenced by the ^3^*J* correlation measurement between the carbon at δ 24.9 (C-6) and deshielded methylene at δ 2.3 (H-4), and ^3^*J* correlation between the carbon at δ 24.9 (C-6) and olefin at δ 5.4 (H-8) (Fig. 1 and 2). ^4^*J* correlation between the neighbouring methylene at δ 2.02 (C-10) of olefin and terminal methyl at δ 0.85 (H-13) confirmed the position of olefin at C-8 and C-9. Although the olefin resonated as a multiplet, the compound was deduced to be in *E* conformation which was more stable compared to *Z* conformation. The spectroscopical data analysis suggest the identity of compound **1** as ethyl-(*E*)-undec-6-enoate.

**Table 1:** ^1^H NMR (CDCl_3_, 600 MHz) and ^13^C NMR (CDCl_3_, 151 MHz) spectral data of compound 1

Position	δ_H_ (ppm)	δ_C_(ppm)	HMBC
1	1.25 (21H, m)	14.3	C-2
2	4.15 (2H, q, *J* = 7.1 Hz)	60.1	C-1, C-3
3	–	174.3	–
4	2.31 (2H, t, *J* = 7.7 Hz))	35.0	C-3, C-5, C-6
5	1.60 (2H, m)	29.1	C-4
6	1.32 (21H, m)	24.9	C-5
7	2.79 (2H, m)	35.0	C-8
8	5.41 (1H, m)	130.0	C-6
9	5.41 (1H, m)	130.0	–
10	2.02 (2H, m)	31.9	C-11, C-9
11	1.35 (21H, m)	29.7	C-13
12	1.25 (21H, m)	2.8	C-11
13	0.85 (4H, t, *J* = 7.1 Hz))	14.3	C-11, C-12

Compound **2** was obtained as reddish-brown crystals (3.9 mg) from the ethyl acetate fraction of *M. oleifera* leaves with HRESIMS molecular ionisation at *m/z* 176.9835 that correspond to [M + 2H]^2+^. Based on the MS data, the compound was deduced to have a molecular weight of 350.24 g/mol with a molecular formula of C_15_H_10_O_10_. From the NMR spectral data (Table 2), compound **2** was found to have a flavanol skeleton with eight hydroxylated carbons, one carbonyl carbon, five quaternary carbons and one methine. Only two doublets signals at δ 6.83 (H-7) and δ 7.89 (H-8) were observed on the ^1^H NMR spectrum. The doublets peaks were *ortho*-positioned to each other with *J* coupling value of 8.6 at A ring of the flavanol. ^3^*J* correlation between the doublets proved the *ortho*-position of the hydrogen. As no other hydrogen signals were observed, it was deduced that all carbons in ring B were attached to hydroxyl groups. In addition, ^3^*J* correlation between δ7.89 (H-8) and δ 168.9 (C-2) confirmed the position of the hydrogen at ring A of the flavanol structure. Based on the spectral data obtained, compound **2** was concluded to be 3,5,6-trihydroxy-2-(2,3,4,5,6-pentahydroxyphenyl)-4*H*-chromen-4-one.

**Table 2:** ^1^H NMR (CD_3_OD, 600 MHz) and ^13^C NMR (CD_3_OD, 151 MHz) spectral data of compound 2

Position	δ_H_ (ppm)	δc (ppm)	COSY	HSQC	HMBC
2	–	168.9	–	–	–
3	–	138.5	–	–	–
4	–	173.8	–	–	–
5	–	162.9	–	–	–
6	–	123.0	–	–	–
7	6.83 (1H, d, *J* = 8.6 Hz)	114.3	H-8	114.3	C-5, C-6, C-8
8	7.89 (1H, d, *J* = 8.7 Hz)	131.4	H-7	131.4	C-2, C-7, C-9, C-10
9	–	161.8	–	–	–
10	–	113.3	–	–	–
1’	–	122.1	–	–	–
2’	–	147.5	–	–	–
3’	–	128.5	–	–	–
4’	–	138.5	–	–	–
5’	–	128.5	–	–	–
6’	–	147.5	–	–	–

Other seven isolated compounds were identified as quercetin (**3**) [26], kaempferol (**4**) [27, 28], β-sitosterol-3-*O*-glucoside (**5**) [29], oleic acid (**6**) [30], glucomoringin (**7**) [31], 2,3,4-trihydroxybenzaldehyde (**8**) [32] and stigmasterol (**9**) [33, 34] based on the MS and NMR comparison with the respective literatures (Fig. 1). 2,3,4-trihydroxybenzaldehyde (**8**) was not included in the bioassay study as the compound was isolated in minute quantity.

### Cell viability

Cell viability assay of *M. oleifera* extracts and the isolated compounds at concentrations of 7.81, 15.62 and 31.25 μg/mL were found not cytotoxic towards the RBL-2H3 cells as presented in Table 3.

**Table 3:** Cell viability of crude extracts on RBL-2H3 cells

	7.81 μg/mL	15.63 μg/mL	31.25 μg/mL
*M. oleifera* leaves	100.37 ± 1.61	101.65 ± 4.45	92.79 ± 6.50
*M. oleifera* seed	96.52 ± 5.50	103.22 ± 2.90	95.07 ± 1.77
*M. oleifera* pod	93.32 ± 13.17	95.72 ± 10.01	92.30 ± 5.18
Ethyl-(*E*)–undec-6-enoate (**MOLH 1**)	94.29 ± 3.73	90.78 ± 7.36	84.30 ± 9.38
Quercetin (**MOLE 1**)	93.96 ± 6.95	82.15 ± 19.95	85.66 ± 17.00
Kaempferol (**MOLE 2**)	108.82 ± 3.69	100.06 ± 9.25	100.18 ± 7.82
3,5,6-trihydroxy-2-(2,3,4,5,6-pentahydroxyphenyl)-4*H*-chromen-4-one (**MOLE 3**)	95.36 ± 5.55	86.50 ± 3.68	83.15 ± 6.98
β-sitosterol-3-*O*-glucoside (**MOLA 1**)	69.54 ± 6.74	64.39 ± 6.29	62.60 ± 11.26
Stigmasterol (**MOPE 1**)	68.78 ± 2.32	54.39 ± 7.15	58.32 ± 5.99
Oleic acid (**MOSE 1**)	82.03 ± 3.24	66.95 ± 4.33	63.63 ± 3.45
Glucomoringin (**MOSA 1**)	77.87 ± 35.88	75.29 ± 10.24	58.63 ± 24.19
Ketotifen fumarate (positive control)	112.74 ± 22.95	100.81 ± 17.00	88.00 ± 3.13

### Inhibitory effect of beta-hexosaminidase release

**Fig. 3:**
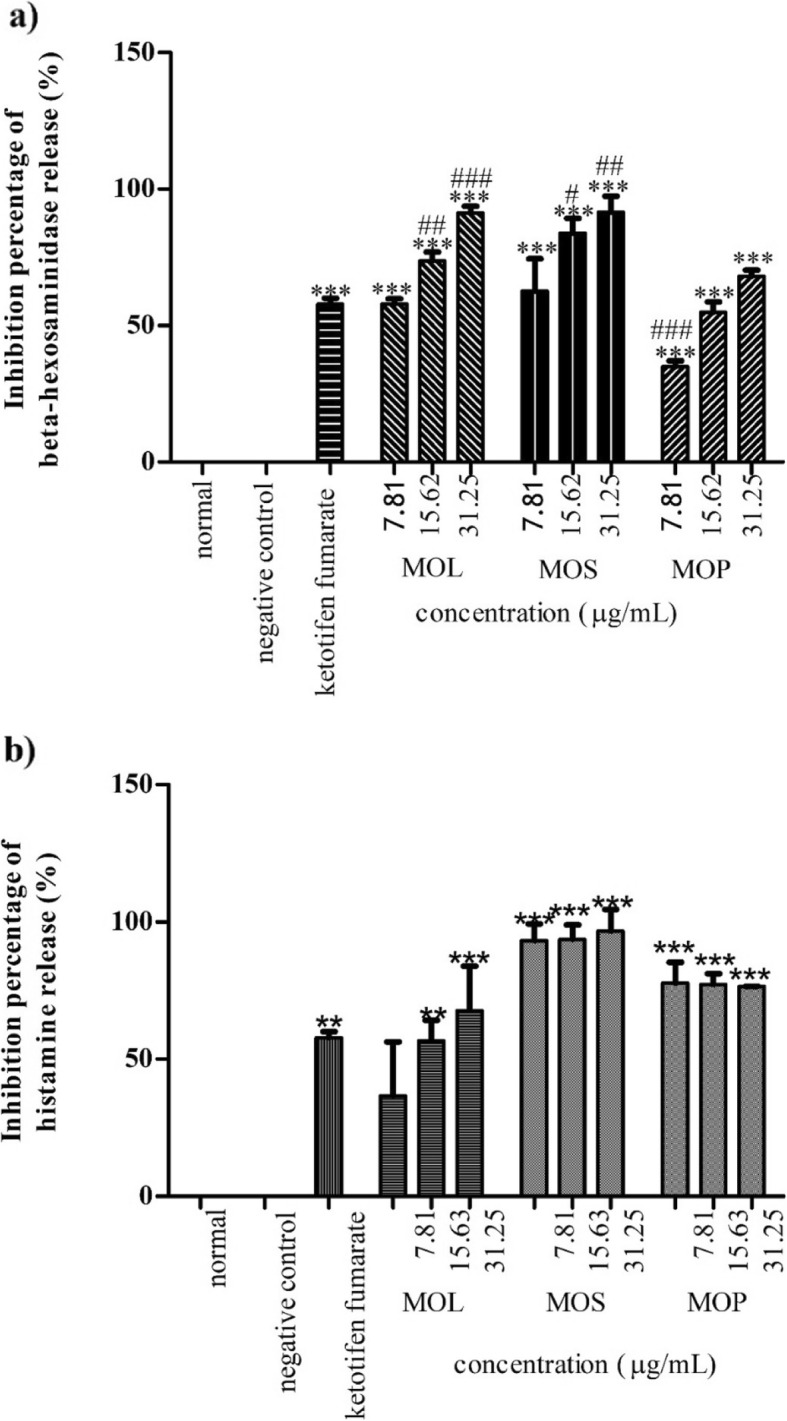
Percentage inhibition of *M. oleifera* crude extracts on **a**) beta-hexosaminidase and **b**) histamine releases. Data are presented as mean ± SEM (*n* = 3) with significant value of **p < 0.01, ***p < 0.001 as compared to positive control and ^#^*p* < 0.05, ^##^p < 0.01, ^###^p < 0.001 as compared to ketotifen fumarate (15.62 μg/mL). MOL: *M. oleifera* leaves, MOS: *M. oleifera* seeds, MOP: *M. oleifera* pods

**Fig. 4:**
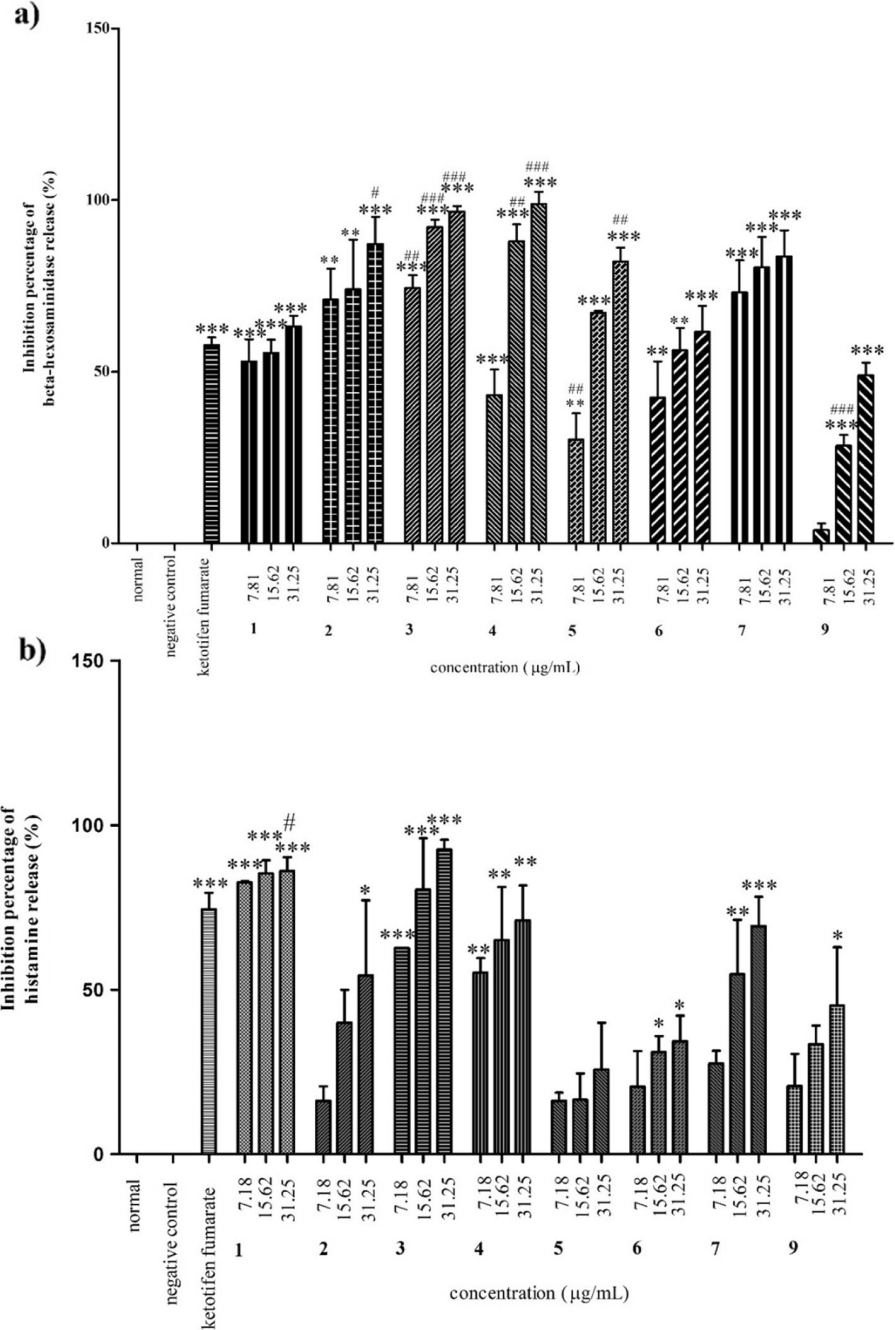
Percentage inhibition of *M. oleifera* test compounds on **a**) beta-hexosaminidase and **b**) histamine releases. Data are presented as mean ± SEM (n = 3) with significant value of *p < 0.05, **p < 0.01, ***p < 0.001 as compared to positive control and and ^#^p < 0.05, ^##^p < 0.01, ^###^p < 0.001 as compared to ketotifen fumarate. Ethyl-(*E*)-undec-6-enoate (1), 3,5,6-trihydroxy-2-(2,3,4,5,6-pentahydroxyphenyl)-4*H*-chromen-4-one (2), Quercetin (3), Kaempferol (4), β-sitosterol-3-*O*-glucoside (5), Oleic acid (6), Glucomoringin (7), Stigmasterol (9)

All extracts (Fig. 3a) and isolated compounds (Fig. 4a) significantly inhibited beta hexosaminidase release in a concentration dependent manner (7.81, 15.62 and 31.25 μg/mL) compared with the positive control, ketotifen fumarate (15.62 μg/mL). Amongst the extracts, the leaves exhibited significantly higher activity with IC_50_ value of 7.17 ± 1.69 μg/mL than ketotifen fumarate with IC_50_ value of 15.19 ± 1.10 μg/mL (Table 4). Four of the isolated compounds from the leaf extract, 3,5,6-trihydroxy-2-(2,3,4,5,6-pentahydroxyphenyl)-4*H*-chromen-4-one (**2**), quercetin (**3**), kaempferol (**4**) and β-sitosterol-3-*O*-glucoside (**5**) exhibited significantly higher inhibitory activity than ketotifen fumarate at varying concentrations of 15.62 and 31.25 μg/mL, and *p*-values (*p* < 0.01 – *p* < 0.001, Fig. 4a). Based on the IC_50_ values presented in Table 4, glucomoringin (**7**) (IC_50_: 10.43 ± 1.51 μM) and quercetin (**3**) (IC_50_: 19.07 ± 7.19 μM) were found to be significantly more active than ketotifen fumarate (IC_50_: 35.70 ± 2.59 μM).

**Table 4:** IC_50_ values of inhibition towards beta-hexosaminidase and histamine release

	IC_50_ values of inhibition	
	Beta-hexosaminidase	Histamine
*M. oleifera* leaf extract	7.17 ± 1.69 μg/mL**	11.66 ± 1.26 μg/mL
*M. oleifera* seed extract	10.68 ± 0.63 μg/mL	5.97 ± 0.84 μg/mL
*M. oleifera* pod extract	14.89 ± 1.25 μg/mL	7.43 ± 1.01 μg/mL
Ethyl-(*E*)–undec-6-enoate (**1**)	82.68 ± 1.98 μM	82.07 ± 13.75 μM
3,5,6-trihydroxy-2-(2,3,4,5,6-pentahydroxyphenyl)-4*H*-chromen-4-one (**2**)	17.70 ± 23.29 μM	44.87 ± 2.13 μM
Quercetin (**3**)	19.07 ± 7.19 μM*	7.77 ± 27.86 μM
Kaempferol (**4**)	29.39 ± 6.26 μM	46.94 ± 10.26 μM
β-sitosterol-3-*O*-glucoside (**5**)	24.93 ± 2.10 μM	–
Oleic acid (**6**)	53.76 ± 8.95 μM	56.05 ± 37.11 μM
Glucomoringin (**7**)	10.43 ± 1.51 μM*	27.22 ± 18.62 μM
Stigmasterol (**9**)	75.92 ± 4.66 μM	38.27 ± 24.48 μM
Ketotifen fumarate (positive control)	15.19 ± 1.10 μg/mL (35.70 ± 2.59 μM)	6.97 ± 0.04 μg/mL (16.38 ± 0.10 μM)

Data are presented as mean ± SEM (*n* = 3) with significance values of *p < 0.05 and **p < 0.01 as compared to positive control.

### Inhibitory effect of histamine release

All extracts significantly inhibited histamine release (Fig. 3b) compared with ketotifen fumarate. All isolated compounds, except β-sitosterol-3-*O*-glucoside (**5**), showed significant inhibitory activity at varying concentrations (Fig. 4b). *M. oleifera* seed extract had better activity with an IC_50_ value of 5.97 ± 0.84 μg/mL than ketotifen fumarate (IC_50_: 6.97 ± 0.04 μg/mL, Table 4). Amongst the isolated compounds, ethyl-(*E*)-undec-6-enoate (**1**), quercetin (**3**) and kaempferol (**4**) significantly inhibited histamine release in a concentration dependent manner (p < 0.01 – *p* < 0.001, Fig. 4b). Oleic acid (**6**) and glucomoringin (**7**) showed significant inhibitory activity at higher concentrations (15.62 and 31.25 μg/mL) (p < 0.05 – p < 0.001, Fig. 4b), whereas 3,5,6-trihydroxy-2-(2,3,4,5,6-pentahydroxyphenyl)-4*H*-chromen-4-one **(2**) and stigmasterol (**9**) significantly inhibited only at the highest concentration (31.25 μg/mL, p < 0.05, Fig. 4b). However, only quercetin (**3**) (IC_50_: 7.77 ± 27.86 μM) was found to be more active than ketotifen fumarate (IC_50_: 16.38 ± 0.10 μM) in inhibiting histamine release.

### Inhibitory effect on cytokines release

**Fig. 5:**
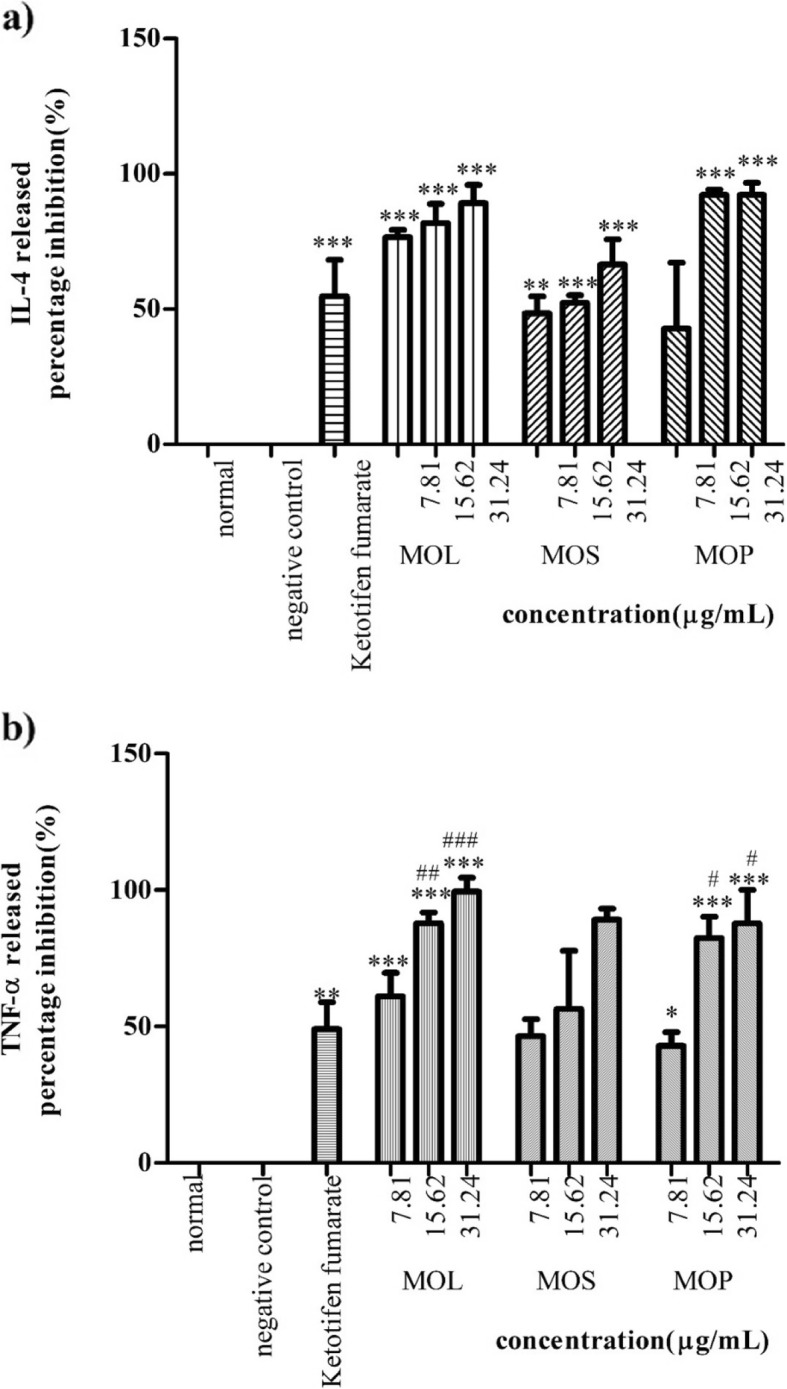
Percentage inhibition of *M. oleifera* crude extracts on **a**) IL-4 and **b**) TNF-α releases. Data are presented as mean ± SEM (n = 3) with significant value of *p < 0.05, **p < 0.01, ***p < 0.001 as compared to positive control and ^#^p < 0.05, ^##^p < 0.01, ^###^p < 0.001 as compared to ketotifen fumarate (15.62 μg/mL). MOL: *M. oleifera* leaves, MOS: *M. oleifera* seeds, MOP: *M. oleifera* pods

**Fig. 6:**
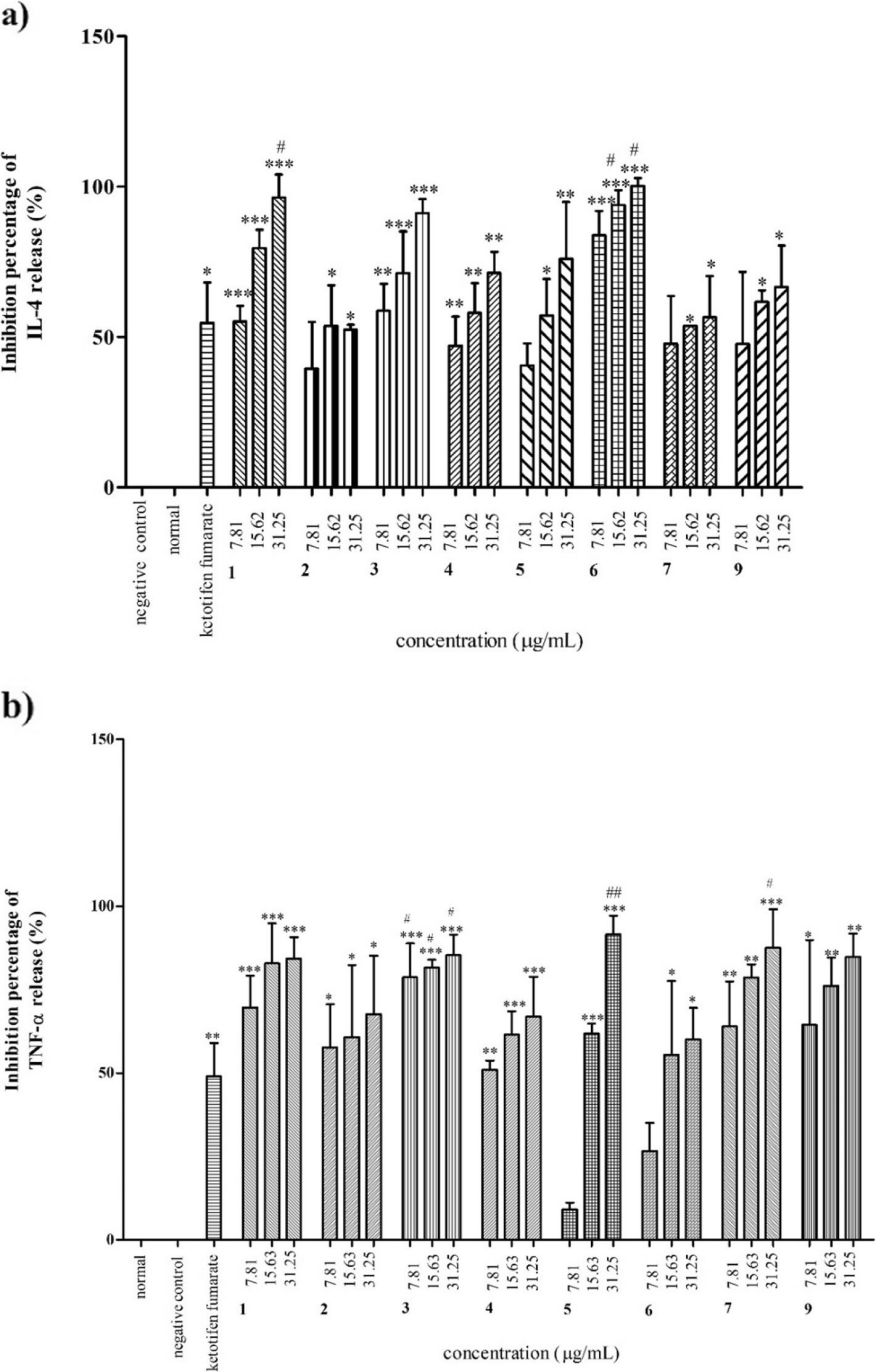
Percentage inhibition of *M. oleifera* test compounds on **a**) IL-4 and **b**) TNF-α releases. Data are presented as mean ± SEM (n = 3) with significant value of *p < 0.05, **p < 0.01, ***p < 0.001 as compared to positive control and and ^#^p < 0.05, ^##^p < 0.01 as compared to ketotifen fumarate. Ethyl-(*E*)-undec-6-enoate (1), 3,5,6-trihydroxy-2-(2,3,4,5,6-pentahydroxyphenyl)-4*H*-chromen-4-one (2), Quercetin (3), Kaempferol (4), β-sitosterol-3-*O*-glucoside (5), Oleic acid (6), Glucomoringin (7), Stigmasterol(9)

Generally, all extracts (Fig. 5a) and the isolated compounds (Fig. 6a) inhibited IL-4 release at varying concentrations, compared with ketotifen fumarate. *M. oleifera* leaf and pod extracts showed higher inhibitory activity with IC_50_ values of 2.32 ± 0.08 and 2.74 ± 0.17 μg/mL, respectively, than ketotifen fumarate (IC_50_: 3.08 ± 0.49 μg/mL) (Table 5). All isolated compounds, except 3,5,6-trihydroxy-2-(2,3,4,5,6-pentahydroxyphenyl)-4*H*-chromen-4-one (**2**) and glucomoringin (**7**), exhibited higher inhibition percentage of IL-4 release at 31.25 μg/mL than ketotifen fumarate (Fig. 6a). However, based on the IC_50_ values (Table 5), β-sitosterol-3-*O*-glucoside (**5**) (IC_50_: 7.33 ± 1.76 μM) and glucomoringin (**7**) (IC_50_: 7.59 ± 0.39 μM) showed the most comparable inhibitory activity to ketotifen fumarate (IC_50_: 7.24 ± 1.16 μM).

**Table 5:** IC_50_ value of inhibition towards IL-4 and TNF-α release

	IC_50_ value of inhibition	
	IL-4	TNF-α
*M. oleifera* leaf extract	2.32 ± 0.08 μg/mL	1.20 ± 0.29 μg/mL
*M. oleifera* seed extract	4.28 ± 0.05 μg/mL	2.52 ± 0.33 μg/mL
*M. oleifera* pod extract	2.74 ± 0.17 μg/mL	2.52 ± 0.15 μg/mL
Ethyl-(*E*)–undec-6-enoate (**1**)	16.91 ± 0.56 μM	9.90 ± 2.31 μM
3,5,6-trihydroxy-2-(2,3,4,5,6-pentahydroxyphenyl)-4*H*-chromen-4-one (**2**)	12.17 ± 0.13 μM	6.45 ± 0.26 μM
Quercetin (**3**)	10.42 ± 0.07 μM	6.24 ± 0.31 μM
Kaempferol (**4**)	14.55 ± 3.24 μM	8.73 ± 0.59 μM
β-sitosterol-3-*O*-glucoside (**5**)	7.33 ± 1.76 μM	5.26 ± 0.00 μM
Oleic acid (**6**)	8.10 ± 0.26 μM	10.11 ± 1.49 μM
Glucomoringin (**7**)	7.59 ± 0.39 μM	3.86 ± 8.26 μM
Stigmasterol (**9**)	10.99 ± 1.57 μM	6.05 ± 16.54 μM
Ketotifen fumarate (positive control)	3.08 ± 0.49 μg/mL (7.24 ± 1.16 μM)	2.91 ± 0.33 μg/mL (6.85 ± 0.77 μM)

For the inhibitory activity of TNF-α release, the extracts of leaves, seeds and pods were found to be more active with IC_50_ values of 1.20 ± 0.29, 2.52 ± 0.33 and 2.52 ± 0.15 μg/mL, respectively, than ketotifen fumarate (IC_50_: 2.91 ± 0.33 μg/mL) (Table 5). Nonetheless, all the isolated compounds also showed higher percentage of inhibitory activity at concentrations of 15.62 and 31.25 μg/mL compared with ketotifen fumarate (Fig. 6b). However, glucomoringin (**7**) (IC_50_: 3.86 ± 8.26 μM) and β-sitosterol-3-*O*-glucoside (**5**) (IC_50_: 5.26 ± 0.00 μM) were found to be even more active than ketotifen fumarate (IC_50_: 6.85 ± 0.77 μM) (Table 5).

## Discussion

*Moringa oleifera* is a commonly used plant that has high nutritional and medicinal values. However, scientific report on its anti-allergic property to support the traditional use is still inadequate. High content of flavonoids and flavanol glucosides in *M. oleifera* such as quercetin, astragalin, kaempferol and rutin could be associated with the anti-allergic potential of the plant [10].

From this study, nine compounds were isolated from the *M. oleifera* ethanol (80%) extracts of various plant parts. Ethyl-(*E*)-2-undec-6-enoate (**1**), 3,5,6-trihydroxy-2-(2,3,4,5,6-pentahydroxyphenyl)-4*H*-chromen-4-one (**2**), quercetin (**3**), kaempferol (**4**) and β-sitosterol-3-*O*-glucoside (**5**) were isolated from the leaves extract. Oleic acid (**6**) and glucomoringin (**7**) were isolated from the seeds extract; while 3,5,6-trihydroxy-2-(2,3,4,5,6-pentahydroxyphenyl)-4*H*-chromen-4-one (**2**), β-sitosterol-3-O-glucoside (**5**), 2,3,4-trihydroxybenzaldehyde (**8**) and stigmasterol (**9**) were isolated from the pod extract. Amongst the compounds isolated, ethyl-(*E*)-2-undec-6-enoate (**1)** and 3,5,6-trihydroxy-2-(2,3,4,5,6-pentahydroxyphenyl)-4*H*-chromen-4-one **(2**) were new compounds isolated in natural product chemistry, whereas 2,3,4-trihydroxybenzaldehyde (**8)** is the first to be reported of its presence in Moringaceae species.

RBL-2H3 cells derived from rat basophils were used to determine the allergic responses in vitro. Basophils have similarities with mast cells based on the composition of the granules and reactions towards allergens [35]. The mast cell degranulation was measured using beta-hexosaminidase and histamine as markers [36]. Upon exposure, the allergens will crosslink with the high-affinity IgE receptor to form IgE-Fc_Ɛ_RI complex triggering the basophils to degranulate and release mediators such as beta-hexosaminidase and histamine [19]. Histamine is a well-known mediator to induce bronchoconstriction and vasodilation in an allergic reaction. However, no significant involvement of beta-hexosaminidase is reported on allergic responses [37].

Early phase of an allergic reaction occurs minutes after allergen sensitisation, releasing histamine and other mediators [19]. Whereas, late phase of the allergic reaction is recognized by the release of cytokines such as IL-13, IL-4, IL-9, IL-5, IFN-γ and TNF-α after 2 to 6 h of sensitisation. The most pivotal cytokines in an allergic reaction are IL-4 and TNF-α. In the presence of IL-4, it will trigger B cell activation and the production of IgE. High amount of IgE will increase the binding site of the allergen on the mast cells, hence induce allergic reactions. TNF-α is responsible for the recruitment of inflammatory cells such as eosinophils, neutrophils, monocytes and macrophages to the site of allergen invasion. This will lead to inflammatory-related allergic responses.

In this study, *M. oleifera* leaf, seed and pod extracts exhibited an anti-allergic activity by inhibiting the early and late phases of allergic reaction. The leaves extract particularly suppressed the release of beta-hexosaminidase, IL-4 and TNF-α more than the positive control, ketotifen fumarate. Nevertheless, it also showed better activity in the late phase of allergic reaction (Table 5). Previous studies also found that this plant suppressed TNF-α level of atopic dermatitis of human keratinocyte cells [38] and LPS-mediated RAW 264.7 macrophage cells [39]. 3,5,6-trihydroxy-2-(2,3,4,5,6-pentahydroxyphenyl)-4*H*-chromen-4-one (**2**), quercetin (**3**), kaempferol (**4**) and β-sitosterol-3-*O*-glucoside (**5**) isolated from the leaves extract inhibited beta-hexosaminidase more actively than ketotifen fumarate. In the early phase of allergic reaction, quercetin (**3**) was found to be more potent than ketotifen fumarate in inhibiting histamine release. In the late phase, 3,5,6-trihydroxy-2-(2,3,4,5,6-pentahydroxyphenyl)-4*H*-chromen-4-one (**2**) and quercetin (**3**) inhibited release of TNF-α, while β-sitosterol-3-*O*-glucoside (**5**) inhibited both IL-4 and TNF-α release more actively than ketotifen fumarate. Flavonoids were reported to have anti-allergic properties by inhibiting histamine and IL-4 release [21]. Three flavonoids isolated from the leaves extract; 3,5,6-trihydroxy-2-(2,3,4,5,6-pentahydroxyphenyl)-4*H*-chromen-4-one (**2**), quercetin (**3**) and kaempferol (**4**), might be responsible for the anti-allergic property due to their ability to stabilise mast cell from degranulation and inhibit the release of IL-4 and TNF-α from IgE-mediated RBL-2H3 cells. Quercetin (**3**) and kaempferol (**4**) have been reported to inhibit mast cells from degranulation by suppressing the calcium influx from extracellular medium [40, 41]. The specific pathway of inhibition was reported to be via the Syk pathway that attenuated the ERK activation and hence inhibited the release of IL-4 and TNF-α release [42, 43]. In addition, quercetin (**3**) and kaempferol (**4**) also inhibited PLCγ and PKC pathways of the IgE-mediated allergic reaction by reducing the expression of 5-lipoxygenase, cyclooxygenase-2 and the production of leukotriene B_4_ and prostaglandin E_2_ [43]. An in-vivo study showed that quercetin (**3**) and kaempferol (**4**) reduced mast-cell count of passive cutaneous anaphylaxis in mice [43]. From a structural activity relationship study, higher number of hydroxyls on ring B of flavanol was found to increase the inhibitory activity of histamine release in RBL-2H3 cells [24]. Quercetin (**3**) with two hydroxyl groups (3′4’-trihydroxy) at the flavanol ring B has higher activity than kaempferol (**4**) with one hydroxyl group (4′-hydroxy group). In contrast, the current study proposed that flavanol with saturated hydroxy group on ring B of 3,5,6-trihydroxy-2-(2,3,4,5,6-pentahydroxyphenyl)-4H-chromen4-one (**2**) decreased the inhibitory activity of the compound.

The findings from this study showed that the extract of *M. oleifera* pods had higher inhibitory activity against beta-hexosaminidase and at the late phase of allergic reaction than ketotifen fumarate. The activity could be associated with its isolated compounds; 3,5,6-trihydroxy-2-(2,3,4,5,6-pentahydroxyphenyl)-4*H*-chromen-4-one (**2**), β-sitosterol-3-*O*-glucoside (**5**) and stigmasterol (**9**). Stigmasterol (**9**) only inhibited TNF-α release in RBL-2H3 cells more significantly than ketotifen fumarate. This is supported by the fact that stigmasterol (**9**) did not inhibit beta-hexosaminidase release from A23187-induced RBL-2H3 cells [44]. However, the compound was found to inhibit scratching and mast cell trafficking in compound 48/80-induced ICR mice [45]. It also decreased TNF-α concentration, ear skin oedema and neutrophil count of Wistar rats induced by 12-*O*-tetradecanoylphorbol-13-acetate (TPA). With an addition of a sugar moeity on the sterol, β-sitosterol-3-*O*-glucoside (**5**) inhibited beta-hexosaminidase, IL-4 and TNF-α release but did not show any significant activity on histamine release. β-sitosterol-3-*O*-glucoside (**5**) might directly inhibit beta-hexosaminidase activity instead of inhibiting mast cell degranulation. In previous studies, β-sitosterol-3-*O*-glucoside (**5**) showed only 7.0% reduction of beta-hexosaminidase release from A23187-induced RBL-2H3 cells [44] and reduced inflammation of xylene-induced oedema in mice [46].

From this study, the *M. oleifera* seeds extract inhibited the release of beta-hexosaminidase, histamine and TNF-α more actively than ketotifen fumarate. It could be attributed to the presence of the isolated compound, glucomoringin (**7**). To the best of the authors’ knowledge, this is the first report of glucomoringin (**7**) and its effect on mast cell stabilising activity and its potential use in inhibiting the late phase of allergic response.

Long chain-containing chemical structure as seen in ethyl-(*E*)-undec-6-enoate (**1**) and oleic acid (**6**) exhibited the weakest mast cell stabilizing activity. Oleic acid (**6**) was reported to weakly inhibit histamine and beta-hexosaminidase release by antigen- and A23187-induced RBL-2H3 cells [47, 48]. In addition, oleic acid only slightly increased intracellular Ca^2+^ that induced cell degranulation [49]. In an in vivo study, peanuts containing high oleic acid was also found to be less allergenic than normal peanuts [50].

## Conclusion

The leaves extract exhibited the highest inhibition against the release of beta-hexosaminidase, IL-4 and TNF-α, while the seed extract inhibited the histamine release the most. Further study revealed that isolated compound such as glucomoringin (**7**) displayed the highest inhibitory activity on beta-hexosaminidase and TNF-α release. Meanwhile, quercetin (**3**) was the most potent against histamine release inhibition while, β-sitosterol-3-*O*-glucoside (**5**) exhibited the highest inhibition on IL-4 release. Overall, this study suggested the anti-allergic property of ethanol (80%) extract of *M. oleifera* leaves, seeds and pods, as well as the isolated compounds by stabilising mast cell from degranulation and inhibiting the early and late phases of allergic reaction in anti-DNP IgE sensitised RBL-2H3 cells induced by DNP-BSA. Two new compounds; ethyl-(*E*)-2-undec-6-enoate (**1)** and 3,5,6-trihydroxy-2-(2,3,4,5,6-pentahydroxyphenyl)-4*H*-chromen-4-one **(2**), were isolated from the leaves extract. However, only the latter compound was active against the beta-hexosaminidase and TNF-α release.

## Data Availability

All-important data regarding the study is included in this published article and any supplementary data is available from the corresponding author upon request.
